# Measures of Food Inadequacy and Cardiovascular Disease Risk in Black Individuals in the US From the Jackson Heart Study

**DOI:** 10.1001/jamanetworkopen.2022.52055

**Published:** 2023-01-23

**Authors:** Rani Zierath, Brian Claggett, Michael E. Hall, Adolfo Correa, Sharrelle Barber, Yan Gao, Sameera Talegawkar, Edith I. Ezekwe, Katherine Tucker, Ana V. Diez-Roux, Mario Sims, Amil M. Shah

**Affiliations:** 1 Division of Cardiovascular Medicine, Brigham and Women’s Hospital, Boston, Massachusetts; 2 University of Mississippi Medical Center, Jackson; 3 Drexel Dornsife School of Public Health, Philadelphia, Pennsylvania; 4 Milken Institute of Public Health at the George Washington University, Washington, DC; 5 Department of Biomedical and Nutritional Sciences, University of Massachusetts, Lowell

## Abstract

**Importance:**

Food insecurity disproportionately affects Black individuals in the US. Its association with coronary heart disease (CHD), heart failure (HF), and stroke is unclear.

**Objective:**

To evaluate the associations of economic food insecurity and proximity with unhealthy food options with risk of incident CHD, HF, and stroke and the role of diet quality and stress.

**Design, Setting, and Participants:**

This cohort study was a time-to-event analysis of 3024 Black adult participants in the Jackson Heart Study (JHS) without prevalent cardiovascular disease (CVD) at visit 1 (2000-2004). Data analysis was conducted from September 1, 2020, to November 30, 2021.

**Exposures:**

Economic food insecurity, defined as receiving food stamps or self-reported not enough money for groceries, and high frequency of unfavorable food stores (>2.5 unfavorable food stores [fast food restaurants, convenience stores] within 1 mile).

**Main Outcomes and Measures:**

The main outcomes were incident CVD including incident CHD, stroke, and HF with preserved ejection fraction and with reduced ejection fraction (HFrEF). During a median follow-up of 13.8 (IQR, 12.8-14.6) years, the associations of measures of food inadequacy with incident CVD (CHD, stroke, and HF) were assessed using multivariable Cox proportional hazards regression models.

**Results:**

Among the 3024 study participants, the mean (SD) age was 54 (12) years, 1987 (66%) were women, 630 (21%) were economically food insecure, and 50% (by definition) had more than 2.5 unfavorable food stores within 1 mile. In analyses adjusted for cardiovascular risk and socioeconomic factors, economic food insecurity was associated with higher risk of incident CHD (hazard ratio [HR], 1.76; 95% CI, 1.06-2.91) and incident HFrEF (HR, 2.07; 95% CI, 1.16-3.70), but not stroke. These associations persisted after further adjustment for diet quality and perceived stress. In addition, economic food insecurity was associated with higher high-sensitivity C-reactive protein and renin concentrations. High frequency of unfavorable food stores was not associated with CHD, HF, or stroke.

**Conclusions and Relevance:**

The findings of this cohort study suggest that economic food insecurity, but not proximity to unhealthy food options, was associated with risk of incident CHD and HFrEF independent of socioeconomic factors, traditional cardiovascular risk factors, diet quality, perceived stress, and other health behaviors. Economic food insecurity was also associated with markers of inflammation and neurohormonal activation. Economic food insecurity may be a promising potential target for the prevention of CVD.

## Key Points

**Question:**

How are measures of food inadequacy associated with risk of incident cardiovascular disease?

**Findings:**

In this cohort study of 3024 Black adult study participants, economic food insecurity, but not proximity to unhealthy food options, was associated with incident coronary heart disease and incident heart failure. Reduced ejection fraction was noted after multivariable adjustment.

**Meaning:**

The findings of this study suggest that economic food insecurity is associated with heart failure with reduced ejection fraction and coronary heart disease and is a promising potential target for the prevention of cardiovascular disease.

## Teaser

This cohort study examines incident cardiovascular disease in Black individuals with economic food insecurity.

## Introduction

Food insecurity is defined as having limited or uncertain access to nutritionally safe and adequate foods that can be acquired in socially acceptable ways.^1^ In 2018, 11.1% of all US households were food insecure (13.9% of all households with children), disproportionally affecting Black, Hispanic, and single-parent households.^2^ The availability of supermarkets in neighborhoods with predominantly Black residents is only 52% of that in neighborhoods comprising predominantly White residents after adjusting for income.^3^ Another dimension of food inadequacy is a lack of physical access to nutritious foods, referred to as food deserts and swamps, which affects approximately 13.5 million people across the US.^4^ In addition, proximity to unfavorable food stores, such as fast food restaurants, has been associated with obesity,^5^ increased blood pressure,^6^ and stroke.^7^ There is abundant evidence that food insecurity and limited physical food access are associated with prevalent hypertension,^8^ diabetes,^9^ and obesity,^10,11^ which are more prevalent in food-insecure communities with a majority of Black residents compared with communities with a majority of White residents.^12^ Both food insecurity and low diet quality are known to be associated with cardiovascular (CV) comorbidities and heart failure (HF) risk factors, suggesting that diet could be a mediating factor to the development of comorbidities among the food insecure.^4,12^ Food insecurity has also been associated with greater psychological stress,^13^ which, in turn, has been associated with CV disease (CVD),^14^ possibly via activation of inflammatory pathways.^15^ However, little is known regarding the extent to which stress may be a factor in the association between food insecurity, inflammation, and incident CVD.

The objective of this analysis was to assess the associations of food insecurity and proximity to unhealthy food options with the risk of incident CV events, including incident HF, coronary heart disease (CHD), and stroke. We also aimed to quantify the relative roles of poor diet quality and greater perceived stress in the associations of food insecurity with these CV outcomes.

## Methods

### Study Population

The Jackson Heart Study (JHS) is a prospective epidemiologic study whose design and methods have previously been described.^16^ A total of 5306 Black adults from Hinds, Madison, and Rankin counties in the Jackson, Mississippi, metropolitan area were initially recruited for a first study visit between 2000 and 2004. The study was approved by the institutional review boards of Jackson State University, University of Mississippi Medical Center, and Tougaloo College; the present study is included within that approval. All participants provided written informed consent; no financial compensation was provided. This analysis included 3024 participants who provided answers to questions regarding food insecurity, had geocoded data, and who were free of prevalent CHD and HF at study visit 1. Data analysis was conducted from September 1, 2020, to November 30, 2021. This study followed the Strengthening the Reporting of Observational Studies in Epidemiology (STROBE) reporting guideline.

### Economic Food Insecurity

Economic food insecurity was assessed via self-report using similar definitions as in prior studies.^17,18^ At the first study visit, participants were asked whether they had received food stamps in the past year. Participants who marked yes were considered economically food insecure for this analysis. Participants were also asked to rate the severity of stress they felt regarding the statement “not enough money for basics such as food.” Participants who marked moderately stressful, stressful, very stressful, or extremely stressful were also considered economically food insecure for this analysis.

### Frequency of Unhealthy Food Options

Unfavorable food stores were defined as convenience stores, bakeries/nuts/candy/ice cream shops, and fast-food establishments (chain and nonchain) and the number of unfavorable food stores within 1 mile was determined by geocoding analysis as previously described (eMethods in Supplement 1 provides detailed description).^19,20^ The number of unfavorable food stores above the median value of 2.5 was considered high.

### Healthy Eating Index

To measure overall diet quality, a Healthy Eating Index (HEI-2015) score was calculated based on the data obtained from a previously validated 158-item Food Frequency Questionnaire administered at visit 1.^21^ The score ranges from 0 to 100, with a higher score indicating better diet quality. The eMethods in Supplement 1 provides further details on the HEI-2015 score.

### Life’s Simple 7 Diet Score

The Life’s Simple 7 diet scores of poor, intermediate, or ideal were determined based on the Food Frequency Questionnaire data at visit 1 as previously defined.^22^ The eMethods in Supplement 1 provides a detailed description.

### Perceived Stress

Overall perceived stress was assessed with the JHS-designed Global Perceived Stress Scale (GPSS), an 8-item continuous measure ranging from 0 to 24, with a higher measure indicating greater stress. This tool evaluates domain-specific stressors experienced during the past year as previously described.^23^ The categories evaluated included jobs, relationships, neighborhood, caregiving, legal, medical, racism and discrimination, and meeting basic needs.^23^

### Assessment of Clinical Covariates, Health Behaviors, Socioeconomic Status, and Social Determinants of Health

Prevalent hypertension was defined by self-reported use of antihypertensive medication or as having a blood pressure 140/90 mm Hg or higher. Diabetes was defined as self-reported use of diabetes medication or a fasting glucose level greater than or equal to 126 mg/dL (to convert to millimoles per liter, multiply by 0.0555) or hemoglobin A_1c_ greater than or equal to 6.5% (to convert to proportion of total hemoglobin, multiply by 0.01).^24^ Chronic kidney disease was defined as having an estimated glomerular filtration rate less than 60 mL/min/1.73 m^2^. Physical activity was assessed with the JHS Physical Activity survey^25^ and categorized as poor, intermediate, and ideal according to Life’s Simple 7 criteria as previously described.^26^ Current smoking was defined as participants who responded yes to the question, “Do you now smoke cigarettes?”^27^

Income was categorized as poor, lower-middle, upper-middle, and affluent based on the US census poverty levels, considering both household income and family size.^28^ Educational level was categorized as less than high school (<12 years), high school graduate or General Educational Development equivalent, some college, or college graduate or greater.^28^ Perceived neighborhood violence, problems, and social cohesion were assessed using dedicated multi-item questionnaires and validated scales created from a principal component analysis and defined at the individual level as described previously.^29,30^ Lifetime discrimination was assessed with the Krieger scale,^28^ and a 0 to 9 score was created based on the number of domains for which unfair treatment was reported, where a higher score is indicative of greater discrimination, as previously described.^28^ The eMethods in Supplement 1 provide detailed information regarding socioeconomic status (SES) and social determinants of health (SDOH).

### Assessment of Pathway Biomarkers

Fasting blood samples were obtained from participants in the supine position and processed in a central laboratory (University of Minnesota) according to standardized protocols described previously.^24^ Methods for measuring B-type natriuretic peptide, renin, high-sensitivity C-reactive protein (hs-CRP), and leptin concentrations in JHS have been previously described^31^ (eMethods in Supplement 1). Insulin resistance was measured by the homeostatic model assessment for insulin resistance using the following formula: fasting plasma insulin (milliunits per liter) × (fasting plasma glucose [millimoles per liter] / 22.5).^31^

### Assessment of Clinical Outcomes

Participants have been followed up for CV events, deaths, and loss to follow-up since the baseline examination in 2000-2004. Cardiovascular events are ascertained by contacting participants annually, surveying discharge lists from local hospitals and death discharge certificates from state vital statistics offices, with subsequent medical record abstraction of eligible CVD events from hospital records and death certificates.^32^ The procedures for CHD, stroke, and HF event adjudication have been previously described in detail^33^ and follow standardized Atherosclerosis Risk in Communities study protocols.^33,34^

A CHD event was defined as a probable or definite myocardial infarction, definite fatal coronary disease, or cardiac procedure.^32,33^ A stroke event was classified as definite or probable based on neuroimaging studies or autopsies in accordance with criteria from the National Survey of Stroke.^35^ An HF event was defined as a probable or definite HF admission with subsequent abstraction and adjudication.^32^ Additional outcomes included HF subtypes: HF with reduced ejection fraction (HFrEF; left ventricular ejection fraction [LVEF]), defined as having an ejection fraction less than 50% at the time of hospitalization, and HF with preserved LVEF (HFpEF), defined as having an ejection fraction greater than or equal to 50% at the time of hospitalization without a previously reduced LVEF. The eMethods in Supplement 1 provide additional details on the ascertainment and classification of CHD, stroke, and HF.

### Statistical Analysis

Logistic regression was used to assess the associations between SDOH and economic food insecurity. Associations of economic food insecurity and incident CV events (HF overall, CHD, HFpEF, HFrEF, and stroke) were assessed using multivariable Cox proportional hazards regression models adjusted for baseline demographic characteristics (age and sex), comorbidities (hypertension, diabetes, body mass index, and estimated glomerular filtration rate), and SES (income and educational level). To assess the potential roles of stress, diet, and other health behaviors (physical activity and smoking), additional models further adjusted for HEI-2015 and GPSS scores in addition to physical activity as well as current and former smoking separately and in combination. Biomarker values were log-transformed to achieve normality. The adjusted geometric means of pathway biomarkers were compared between economically food insecure and non–food insecure groups. Adjustment covariates included demographic characteristics, CV risk factors, and SES factors. Similar analyses were performed assessing the associations of physical food access with risk of incident cardiovascular events. Fine and Gray proportional subhazards models were used to assess the competing risk of death with all CVD outcomes.^36^

A 2-sided *P* value <.05 was considered statistically significant. All analyses were performed using Stata, version 14 (StataCorp LLC).

## Results

The study sample was composed of 3024 adults free of HF and CHD at baseline who had adequate food store and food insecurity data (eFigure in Supplement 1). The 2085 adults excluded from this analysis were older, more likely male, with a higher prevalence of CV risk factors, including hypertension, diabetes, chronic kidney disease, current smoking, and worse physical activity. The excluded population also had higher event rates for all cardiovascular outcomes, including incident HF, HFpEF, HFrEF, CHD, and stroke, compared with the included population (eTable 1 in Supplement 1). The mean (SD) age of the study population was 54 (12) years, 1037 participants were men (34%), 1987 (66%) were women, 1585 (52%) had hypertension, and 623 (21%) had diabetes. Compared with those who were not economically food insecure, participants experiencing economic food insecurity were younger, more likely to be women, and had a higher prevalence of hypertension and greater BMI (Table 1). Participants experiencing food insecurity reported higher perceived stress than those not experiencing food insecurity (mean [SD] GPSS score, 7.8 [5.1] vs 4.5 [3.8]; *P* < .001) and had lower diet quality (mean [SD] HEI-2015 score, 45.8 [10.5] vs 47.7 [10.5]; *P* < .001). Economic food insecurity was also associated with several other SDOH, including lower income, lower educational attainment, greater perceived lifetime discrimination, greater reported neighborhood problems and violence, but also with greater neighborhood social cohesion. In models adjusted for demographic characteristics, comorbidities, and SES factors, economic food insecurity remained associated with greater lifetime discrimination (odds ratio [OR], 1.27; 95% CI, 1.12-1.43), lower income group (OR, 2.11; 95% CI, 1.86-2.40), and higher neighborhood social cohesion (OR, 1.17; 95% CI,1.04-1.32) (Table 2).

**Table 1.** Baseline Characteristics of Study Population Overall and Stratified by Economic Food Insecurity Status

Characteristic	No. (%)			*P* value
	Overall (n = 3024)	Non–food insecure (n = 2394)	Food insecure (n = 630)	
Demographic				
Age, mean (SD)	54 (12)	55 (12)	50 (13)	<.001
Sex				
Female	1987 (66)	1514 (63)	473 (75)	<.001
Male	1037 (34)	880 (37)	157 (25)	
Comorbidities				
BMI, mean (SD)	31.7 (7.1)	31.2 (6.6)	33.3 (8.6)	<.001
Hypertension	1585 (52)	1246 (52)	339 (54)	.43
Diabetes	623 (21)	471 (20)	152 (24)	.02
CKD	132 (4)	84 (4)	48 (8)	<.001
eGFR, mean (SD), mL/min/1.73 m^2^	96.1 (20.5)	94.9 (19.9)	100.4 (22.0)	<.001
Health behaviors				
Smoking categorization				
Current smoker	328 (11)	223 (9)	105 (17)	<.001
Quit <12 mo ago	37 (1)	24 (1)	13 (2)	
Quit >12 mo ago/never smoked	2618 (88)	2112 (90)	506 (81)	
Physical activity				
Poor	1378 (46)	1061 (44)	317 (50)	.01
Intermediate	1016 (34)	813 (34)	203 (32)	
Ideal	630 (21)	520 (22)	110 (17)	
Diet and stress				
Perceived stress, mean (SD)	5.2 (4.3)	4.5 (3.8)	7.8 (5.1)	<.001
Life’s Simple 7 Diet Score				
Poor	1878 (68)	1466 (66)	412 (74)	.002
Intermediate	848 (31)	706 (32)	142 (25)	
Ideal	38 (1)	34 (2)	4 (1)	
Healthy Eating Index score, mean (SD)	47.3 (10.5)	47.7 (10.5)	45.8 (10.5)	<.001
Socioeconomic status and SDOH				
Neighborhood % below poverty limit, mean (SD)	0.23 (0.13)	0.23 (0.13)	0.25 (0.12)	<.001
Income categorization				
Poor	331 (13)	161 (8)	170 (32)	<.001
Lower-middle	579 (22)	433 (21)	146 (27)	
Upper-middle	808 (31)	663 (32)	145 (27)	
Affluent	896 (34)	819 (39)	77 (14)	
Educational level categorization				
<High school	383 (13)	277 (12)	106 (17)	<.001
High school graduate/GED	569 (19)	422 (18)	147 (23)	
Vocational school, trade school, college	2070 (68)	1693 (71)	377 (60)	
Lifetime discrimination, median (IQR)^a^	3 (1-5)	3 (1-4)	3 (2-5)	.007
Neighborhood problems, median (IQR)	1.56 (1.37-1.71)	1.56 (1.37-1.71)	1.58 (1.43-1.72)	<.001
Neighborhood social cohesion, median (IQR)	3.03 (2.93-3.12)	3.03 (2.94-3.12)	2.99 (2.91-3.07)	<.001
Neighborhood violence, median (IQR)	1.26 (1.15-1.32)	1.24 (1.15-1.32)	1.28 (1.18-1.34)	<.001

Abbreviations: BMI, body mass index (calculated as weight in kilograms divided by height in meters squared); CKD, chronic kidney disease; eGFR, estimated glomerular filtration rate; GED, General Educational Development; SDOH, social determinants of health.

^a^ Score range is 0 to 9, with a higher score indicating greater discrimination.

**Table 2.** Associations of Economic Food Insecurity With Neighborhood Variables, Discrimination, and SES Factors

Variable	Model covariates					
	Demographic characteristics^a^		Demographic characteristics, comorbidities^b^		Demographic characteristics, comorbidities, SES^c^	
	OR (95% CI)	*P* value	OR (95% CI)	*P* value	OR (95% CI)	*P* value
Standardized neighborhood violence	1.37 (1.24-1.52)	<.001	1.35 (1.22-1.50)	<.001	1.13 (1.00-1.27)	.05
Standardized % below poverty limit	1.31 (1.18-1.46)	<.001	1.30 (1.17-1.44)	<.001	1.05 (0.93-1.19)	.46
Standardized educational level	0.68 (0.61-0.75)	<.001	0.68 (0.62-0.76)	<.001	0.88 (0.78-1.00)	.06
Standardized discrimination	1.11 (1.00-1.23)	.044	1.12 (1.01-1.24)	.034	1.27 (1.12-1.43)	<.001
Standardized income group	2.21 (1.97-2.48)	<.001	2.19 (1.95-2.46)	<.001	2.11 (1.86-2.40)	<.001
Standardized neighborhood problems	1.36 (1.22-1.50)	<.001	1.33 (1.20-1.48)	<.001	1.08 (0.96-1.23)	.20
Standardized neighborhood social cohesion	1.40 (1.26-1.54)	<.001	1.37 (1.24-1.52)	<.001	1.17 (1.04-1.32)	.008

Abbreviations: OR, odds ratio; SES, socioeconomic status.

^a^ Include age and sex.

^b^ Include hypertension, diabetes, and body mass index.

^c^ Include comorbidities, income level, and educational attainment.

### Economic Food Insecurity

Over a median follow-up period of 13.8 (IQR, 12.8-14.6) years, 123 participants experienced a CHD event, 195 developed HF (88 HFrEF, 88 HFpEF, and 19 with unknown LVEF), and 104 experienced a stroke. In models adjusted for demographic characteristics (age and sex), economic food insecurity was associated with a heightened risk of incident CHD, incident HF, and incident HFrEF in particular (Figure; eTable 2 in Supplement 1). No association was observed between economic food insecurity and incident HFpEF or incident stroke. After further adjustment for comorbidities (hypertension, diabetes, and body mass index) and SES (income and educational level), economic food insecurity remained associated with a higher risk of incident coronary disease (hazard ratio [HR], 1.76; 95% CI, 1.06-2.91) and incident HFrEF (HR, 2.07; 95% CI, 1.16-3.70), with minimal attenuation of the effect estimate (eTable 2 in Supplement 1). After further adjustment for physical activity, smoking, diet (Life’s Simple 7 diet score or HEI-2015 score), and stress (GPSS score) individually and together, the association of economic food insecurity with incident CHD and incident HFrEF persisted with minimal attenuation of the effect estimate (Figure; eTable 3 in Supplement 1). The effect estimates of these associations did not change substantially when accounting for the competing risk of death (eTable 4 in Supplement 1). In addition, associations with incident HFrEF persisted in analyses censoring participants with an interval CHD event and when assigning HF events with an unknown EF as HFpEF or HFrEF (eTable 5 and eTable 6 in Supplement 1). In analyses adjusted for demographic characteristics, CV risk factors, and SES factors, participants experiencing economic food insecurity had significantly higher concentrations of renin and hs-CRP compared with those who were categorized as non–food insecure (Table 3).

**Figure.**
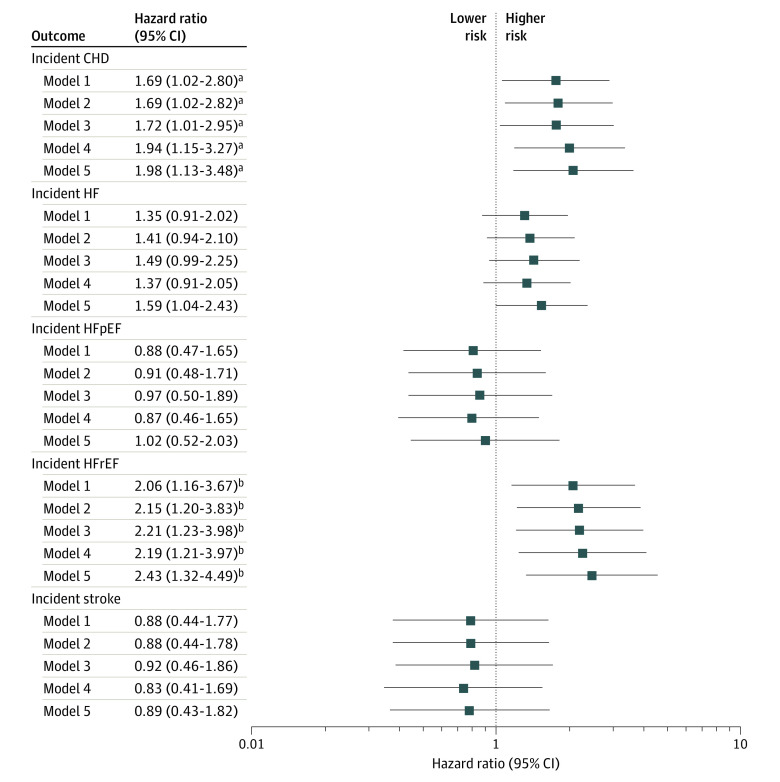
Association of Economic Food Insecurity With Incident Coronary Heart Disease (CHD), Heart Failure (HF) Overall, Heart Failure With Preserved Ejection Fraction (HFpEF), Heart Failure With Reduced Ejection Fraction (HFrEF), and Stroke Model 1 was adjusted for age, sex, hypertension, diabetes, body mass index (BMI), income, and educational level. Model 2 was adjusted for model 1 plus health behaviors (physical activity and smoking). Model 3 was adjusted for model 1 plus diet. Model 4 was adjusted for model 1 plus stress. Model 5 was adjusted for model 1 plus physical activity, smoking, diet, and stress. ^a^*P* < .05. ^b^*P* < .05.

**Table 3.** Biomarker Values Displayed in the Overall Population and Stratified by Food Insecurity Status as Adjusted Geometric Means

Biomarker	Overall, mean (95% CI)	Adjusted, mean (95% CI)^a^		
		Non–food insecure	Food insecure	*P* value
BNP, pg/mL	7.3 (7.0-7.7)	8.0 (7.5-8.4)	7.5 (6.7-8.4)	.38
Fasting insulin, μIU/mL	14.8 × 10^6^ (14.5 × 10^6^-15.1 × 10^6^)	15.4 × 10^6^ (15.1 × 10^6^ −15.8 × 10^6^)	15.2 × 10^6^ (14.5 × 10^6^-16.0 × 10^6^)	.61
HOMA-IR	2.3 (2.3-2.4)	3.1 (3.0-3.2)	3.0 (2.8-3.1)	.19
CRP, mg/dL	0.28 (0.27-0.30)	0.30 (0.28-0.32)	0.34 (0.30-0.38)	.02
Leptin, ng/mL	19.7 (19.1-20.4)	27.9 (27.0-28.8)	26.8 (25.4-28.2)	.18
Renin, ng/mL/h	0.59 (0.56-0.63)	0.60 (0.55-0.65)	0.74 (0.62-0.86)	.03

Abbreviations: BNP, B-type natriuretic peptide; CRP, C-reactive protein; HOMA-IR, homeostatic model assessment for insulin resistance.

SI conversion factors: To convert BNP to nanograms per liter, multiply by 1; CRP to milligrams per liter, multiply by 10; and fasting insulin to picomoles per liter, multiply by 6.945.

^a^ Covariates for adjusted geometric means include age, sex, hypertension, diabetes, body mass index, income, and educational level.

### Unfavorable Food Stores

Compared with those who had less than 2.5 unfavorable food stores within 1 mile, participants who had more than 2.5 unfavorable food stores within 1 mile of their home were older, more likely to be women, and had a higher prevalence of hypertension and diabetes (eTable 7 in Supplement 1). Perceived stress was moderately higher and overall diet quality was similar between those who had more than 2.5 unfavorable food stores and less than 2.5 within 1 mile (mean [SD] GPSS score, 5.4 [4.6] vs 5.0 [4.0], *P* = .04; HEI-2015 score, 47.4 [10.7] vs 47.1 [10.3], *P* = .56). In multivariable Cox proportional hazards regression models adjusted for participant demographic characteristics, having more than 2.5 unfavorable food stores within 1 mile was not associated with any CV outcome (Table 4). Similarly, no associations were observed after further adjustment for clinical comorbidities and SES. Similar findings were observed in analyses comparing those with number of unfavorable food stores within 1 mile in the upper quartile (>7.1 unfavorable food stores) compared with the other quartiles and in analyses modeling the number of unfavorable food stores within 1 mile as a continuous variable (eTable 8 and eTable 9 in Supplement 1).

**Table 4.** Association of Proximity to Unfavorable Food Stores With Incident HF, HFpEF, HFrEF, CHD, and Stroke

Variable	No.	Person-time	Events (%)	Event rate (95% CI per 100 person-years)	Regression model					
					Unadjusted		Adjusted for demographic characteristics^a^		Adjusted for demographic characteristics, comorbidities, SES^b^	
					HR (95% CI)	*P* value	HR (95% CI)	*P* value	HR (95% CI)	*P* value
**Incident HF**										
<2.5 Food stores	1506	32.3	74 (5)	2.3 (1.8-2.9)	1.35 (1.01-1.80)	.04	1.06 (0.77-1.46)	.72	1.07 (0.74-1.54)	.71
>2.5 Food stores	1512	34.6	121 (9)	3.5 (2.9-4.2)						
**Incident HFpEF**										
<2.5 Food stores	1506	32.3	26 (2)	0.8 (0.5-1.2)	2.00 (1.26-3.16)	.003	1.22 (0.75-1.99)	.43	1.18 (0.69-2.02)	.55
>2.5 Food stores	1512	34.6	62 (4)	1.8 (1.4-2.3)						
**Incident HFrEF**										
<2.5 Food stores	1506	32.3	40 (3)	1.2 (0.9-1.7)	0.98 (0.64-1.50)	.91	0.94 (0.59-1.52)	.81	1.03 (0.59-1.80)	.92
>2.5 Food stores	1512	34.6	48 (3)	1.4 (1.0-1.8)						
**Incident CHD**										
<2.5 Food stores	1506	188.6	52 (4)	0.3 (0.2-0.4)	1.39 (0.97-1.98)	.07	1.18 (0.78-1.78)	.44	1.05 (0.66-1.67)	.84
>2.5 Food stores	1512	186.4	71 (5)	0.4 (0.3-0.5)						
**Incident stroke**										
<2.5 Food stores	1506	184.7	41 (3)	0.2 (0.1-0.3)	1.57 (1.03-2.38)	.04	1.12 (0.71-1.77)	.64	1.08 (0.64-1.83)	.77
>2.5 Food stores	1512	178.6	63 (4)	0.3 (0.3-0.4)						

Abbreviations: CHD, coronary heart disease; HF, heart failure; HFpEF, heart failure with preserved ejection fraction; HFrEF, heart failure with reduced ejection fraction; HR, hazard ratio; SES, socioeconomic status.

^a^ Include age and sex.

^b^ Include hypertension, diabetes, body mass index, estimated glomerular filtration rate, income level, and educational attainment.

## Discussion

This study of the association of food insecurity with CVD risk in 3024 Black adults from Jackson, Mississippi, has 3 major novel findings. First, economic food insecurity was a risk factor for incident CHD and incident HFrEF. These associations persisted even after accounting for traditional cardiovascular risk factors and SES, including income, suggesting that the impact of food insecurity extends beyond economic disadvantage. Second, participants experiencing economic food insecurity had greater systemic inflammation, reflected in higher hs-CRP concentrations, and had greater neurohormonal activation, reflected in higher circulating renin concentrations—both pathways that are implicated in atherosclerosis and HF pathobiologic pathways. Third, the risk associated with economic food insecurity was not accounted for by physical activity, smoking, diet quality, or perceived stress, suggesting alternative factors linking economic food insecurity to CVD risk. Notably, frequency of unhealthy foods in this analysis was not associated with risk of CVD. Together, these findings support economic food insecurity, which disproportionately impacts Black communities, as a risk factor for CHD and HFrEF and a potential factor in well-documented racial disparities in CV health.

Food insecurity disproportionally impacts Black individuals in the US. A US Department of Agriculture report examining trends in food insecurity during 15 years reported that rates of food insecurity for non-Hispanic Black households were twice those of non-Hispanic White households.^37^ In a study examining poor physical food access and premature CVD-associated death across Atlanta, 85% of all premature deaths occurred among Black individuals.^17^ Food insecurity has been associated with heightened risk of CV mortality,^17^ although data relating food insecurity to specific CVD are more limited. Existing studies are largely restricted to cross-sectional associations or studies in persons with prevalent disease. Previous studies from the National Health and Nutrition Examination Survey reported a higher age-adjusted prevalence of food insecurity among persons with prevalent CHD, compared with those free of CHD.^38,39^ Conversely, a study of more than 40 000 adults living below the poverty limit noted a higher predicted prevalence of CHD with worsening severity of food insecurity.^40^ Consistently, our study provides data that economic food insecurity in persons free of prevalent CHD at baseline is associated with a 76% higher risk of CHD development over 14 years of follow-up after adjustment for demographic characteristics, comorbidities, and SES.

Less is known regarding the association between food insecurity and the development of HF. Data from the National Health and Nutrition Examination Survey noted a higher age-standardized prevalence of food insecurity among persons with HF compared with those without HF.^39^ Living in a food desert has also been associated with a higher risk of recurrent HF hospitalization among persons with prevalent HF.^41^ However, these analyses are limited by potential reverse causation, given the economic burden associated with chronic medical conditions, such as HF, in the US.^42^ To our knowledge, our study is one of the first to relate food insecurity to risk of developing HF and to assess for differential associations with risk of developing HFrEF or HFpEF. We observed a 2-fold higher risk of HFrEF among individuals experiencing economic food insecurity and no association with HFpEF. This association persisted after censoring interval CHD events, suggesting that the association between food insecurity and HFrEF is not mediated by CHD or myocardial infarction.

The mechanisms relating food insecurity to CHD and HFrEF risk independent of SES and CV comorbidities are unclear. Participants experiencing economic food insecurity had higher hs-CRP concentrations, suggesting an association with greater systemic inflammation. The proportion of study participants with an abnormal hs-CRP concentration (>5 mg/dL) was 39% among those experiencing food insecurity and 36% in the non–food insecure group. This finding is consistent with other large studies in multiethnic populations noting higher hs-CRP levels among adults living in food deserts^43^ and reporting an association between these 2 factors.^44^ This finding is also consistent with the wealth of literature linking inflammation to allostatic load, which is one mechanism connected to the health consequences of unfavorable SDOH.^45^ C-reactive protein concentration is not associated with the development of CVD but is a valid marker of inflammation. Participants experiencing economic food insecurity also showed higher concentrations of circulating renin, a marker of increased activity of the renin-angiotensin-aldosterone system (RAAS), despite a similar prevalence of hypertension in the economically food insecure vs non–food insecure groups (54% vs 52%; *P* = .43). While we are not aware of prior descriptions of the association of food insecurity with RAAS activation, heightened RAAS activity is associated with greater psychological stress,^46^ which is linked with food insecurity^13^ and associated SDOH including discrimination,^47^ SES,^48^ and neighborhood disadvantage.^48^ Both systemic inflammation and neurohormonal activation are central pathophysiologic processes that underly atherosclerosis and HF development. These findings are therefore concordant with—and supportive of—the observed associations of economic food insecurity with incident CHD and HFrEF. The reasons for the lack of association of economic food insecurity with incident HFpEF and stroke are unclear, as inflammation has also been implicated in the pathobiologic aspects of both outcomes. However, these findings suggest that specific social determinants of health my differentially increase the risk for distinct CV outcomes.

The potential triggers for greater systemic inflammation and neurohormonal activation among the economically food insecure population are not defined. We hypothesized that worse diet quality and greater perceived stress may result from food insecurity and partially mediate associations with adverse CV outcomes. Both diet quality and perceived stress have been associated with inflammation and neurohormonal activation,^15,46,49^ and with risk for CVD.^8,14^ Consistent with prior studies, economic food insecurity was associated with greater perceived stress and worse diet quality in our analysis.^4,12,13^ However, adjusting for perceived stress and diet quality did not appreciably impact the association of economic food insecurity with incident CHD and HFrEF. These findings suggest that other factors outside of diet and stress mediate the associations between food insecurity, CHD, and HF.

Economic food insecurity is associated with lower income status, which is not surprising given that income is inherent in the definition of economic food insecurity.^1^ Despite this robust association, the association of economic food insecurity with CHD and HFrEF was independent of income and educational attainment in our analysis. Economic food insecurity was also associated with several additional SDOH, including lifetime discrimination, consistent with prior studies,^50^ and neighborhood problems or violence. The associations between neighborhood characteristics and economic food insecurity are largely unexplored. Given the interrelatedness of several SDOH, many adults are burdened by more than 1 unfavorable SDOH and a greater number of unfavorable factors has been associated with greater CV risk.^51^ Further research is needed to understand the interrelations of food insecurity with different SDOH and psychosocial responses and the extent to which they may mediate and/or modulate the association of food insecurity with CVD.

Proximity to unhealthy food options, a measure of physical food access, was not associated with incident CVD in this study. The existing data regarding poor physical access to food and CVD are complex. Similar to our study, the META-Health and Predictive Health studies, which enrolled adults residing in Atlanta, found a higher prevalence of CV risk factors among those living in a food desert (a measure of low healthy food access and low income), but no association between living in a food desert and 10-year CV risk.^43^ In contrast, in another study of Atlanta adults, food access score—a measure that does not incorporate information on income—was associated with premature CV death.^17^ Furthermore, among persons with prevalent CVD, food deserts are associated with worse prognosis.^38^ Although prior studies have established the association between the frequency of unhealthy food options and the prevalence of CV risk factors,^52^ it is possible that this measure of physical food access does not adequately capture the nature of one’s food environment compared with the more commonly used food desert measures.

Our findings are observational, and further prospective intervention studies are needed to define whether intervening on economic food insecurity will yield reductions in risk of CHD and/or HFrEF. However, our findings provide a rationale to expect that targeting food insecurity could reduce incident CHD and HF and help mitigate the marked racial disparity in the burden of CVD in the US.^53^ Addressing food insecurity may be accomplished through policy changes at the federal and local levels, including (but not limited to) the expansion of federal food resources such as the Supplemental Nutrition Assistance Program, the implementation of community food resource programs, increasing opportunities for employment, and increased implementation of reimbursement programs for food insecurity screening, such as the Comprehensive Hospital Increased Reimbursement Program through Texas Health and Human Services.^50,54^ While these interventions are outside the purview of most CV clinicians, diagnosing this risk marker to help motivate the necessary policy prescriptions is not.^50^ Despite this, one study reported that screening for food insecurity in physician practices is only 30% and in hospitals is only 40%.^55^

### Limitations

This study has several limitations. Measures of diet quality, stress, and economic food insecurity were derived from self-report and are subject to misclassification. This study used definitions for economic food insecurity and physical food environment that are different from the more commonly used United States Department of Agriculture–derived food insecurity and food desert measures, which may lead to the misclassification of participants as food insecure. However, the definitions we used are similar to those applied by other high-quality community studies to measure economic and physical aspects of food access.^11,17,18,52^ Furthermore, the food insecurity measures in this study were only considered at baseline, and therefore do not account for the potential for food insecurity status to change over time. The study population was restricted to Black adults living in a southern metropolitan area and may not be generalizable to Black individuals living in other areas of the US. Furthermore, ascertainment of HF events after visit 1 did not begin until January 2005. However, this delay in the surveillance of HF events may protect this analysis from reverse causality owing to subclinical HF at visit 1. In addition, there were 19 incident HF events that occurred during follow-up with an unknown EF at the time of the event. However, a sensitivity analysis assigning the unknown cases as either HFpEF or HFrEF noted similar findings to our primary analysis. Although the differences in adjusted geometric means for markers of inflammation and neurohormonal activation were greater in individuals experiencing food insecurity compared with those who were not, the mean values of these biomarkers within each group were not above the reference range. This analysis also demonstrated associations between food insecurity and other SDOH, including discrimination, neighborhood social cohesion, and income. Due to the interrelatedness and often cumulative nature of these factors, we are limited in our ability to disentangle the impact of food insecurity from that of other associated SDOH, raising the possibility that the associations we report remain at least partly confounded by unmeasured neighborhood environmental factors or individual-level social circumstances.

## Conclusions

This analysis from a large epidemiologic cohort study of Black individuals in the US suggests that economic food insecurity is a risk factor for incident CHD and incident HFrEF, independent of socioeconomic measures (eg, income, educational attainment) and traditional CV risk factors. Greater systemic inflammation and neurohormonal activation were present in participants experiencing economic food insecurity. These findings support economic food insecurity, which disproportionately affects Black communities, as an important factor in the well-documented racial disparities in CV health, and as a promising potential target for intervention.

## Notes

Supplement 1. **eMethods.** Detailed Methods **eTable 1.** Baseline Characteristics of the Study Population Included and Excluded From the Study **eTable 2.** Association of Economic Food Insecurity With Incident HF, HFpEF, HFrEF, CHD, and Stroke **eTable 3.** Association of Economic Food Insecurity With Incident HF, HFpEF, HFrEF, and CHD Adjusted for Diet Quality and Stress **eTable 4.** Association of Economic Food Insecurity With Incident HF, HFpEF, HFrEF, CHD, and Stroke Taking Into Account the Competing Risk of Death **eTable 5.** Association of Economic Food Insecurity With Incident HF, HFpEF, HFrEF, CHD, and Stroke Censoring Interim MI Events **eTable 6.** Association of Economic Food Insecurity With Incident HFpEF and HFrEF, With Unknown EF Assigned as HFpEF or HFrEF **eTable 7.** Baseline Characteristics of Study Population Overall and Stratified by Unfavorable Food Stores (Greater or Less Than 2.5 Food Stores Within 1 Mile) **eTable 8.** Association of Proximity to Unfavorable Food Stores With Incident HF, HFpEF, HFrEF, CHD, and Stroke **eTable 9.** Association of High Frequencies of Unfavorable Food Stores With Incident HF, HFpEF, HFrEF, CHD, and Stroke **eFigure.** Study Population Flow Diagram

Supplement 2.
Data Sharing Statement
