# Supplementary material for: Measures of Food Inadequacy and Cardiovascular Disease Risk in Black Individuals in the US From the Jackson Heart Study
Source: JAMA Netw Open. 2023 Jan 23;6(1):e2252055. doi: 10.1001/jamanetworkopen.2022.52055 (PMC9871801; doi:10.1001/jamanetworkopen.2022.52055)
Supplement: Supplement 1. — eMethods. Detailed Methods eTable 1. Baseline Characteristics of the Study Population Included and Excluded From the Study eTable 2. Association of Economic Food Insecurity With Incident HF, HFpEF, HFrEF, CHD, and Stroke eTable 3. Association of Economic Food Insecurity With Incident HF, HFpEF, HFrEF, and CHD Adjusted for Diet Quality and Stress eTable 4. Association of Economic Food Insecurity With Incident HF, HFpEF, HFrEF, CHD, and Stroke Taking Into Account the Competing Risk of Death eTable 5. Association of Economic Food Insecurity With Incident HF, HFpEF, HFrEF, CHD, and Stroke Censoring Interim MI Events eTable 6. Association of Economic Food Insecurity With Incident HFpEF and HFrEF, With Unknown EF Assigned as HFpEF or HFrEF eTable 7. Baseline Characteristics of Study Population Overall and Stratified by Unfavorable Food Stores (Greater or Less Than 2.5 Food Stores Within 1 Mile) eTable 8. Association of Proximity to Unfavorable Food Stores With Incident HF, HFpEF, HFrEF, CHD, and Stroke eTable 9. Association of High Frequencies of Unfavorable Food Stores With Incident HF, HFpEF, HFrEF, CHD, and Stroke eFigure. Study Population Flow Diagram [file jamanetwopen-e2252055-s001.pdf]

Supplementary Online Content

Zierath R, Claggett B, Hall ME, et al. Measures of food inadequacy and cardiovascular disease risk in Black individuals in the US from the Jackson Heart Study. *JAMA Netw Open*. 2023;6(1):e2252055. doi:10.1001/jamanetworkopen.2022.52055

eMethods. Detailed Methods

eTable 1. Baseline Characteristics of the Study Population Included and Excluded From the Study

eTable 2. Association of Economic Food Insecurity With Incident HF, HFpEF, HFrEF, CHD, and Stroke

eTable 3. Association of Economic Food Insecurity With Incident HF, HFpEF, HFrEF, and CHD Adjusted for Diet Quality and Stress

eTable 4. Association of Economic Food Insecurity With Incident HF, HFpEF, HFrEF, CHD, and Stroke Taking Into Account the Competing Risk of Death

eTable 5. Association of Economic Food Insecurity With Incident HF, HFpEF, HFrEF, CHD, and Stroke Censoring Interim MI Events

eTable 6. Association of Economic Food Insecurity With Incident HFpEF and HFrEF, With Unknown EF Assigned as HFpEF or HFrEF

eTable 7. Baseline Characteristics of Study Population Overall and Stratified by Unfavorable Food Stores (Greater or Less Than 2.5 Food Stores Within 1 Mile)

eTable 8. Association of Proximity to Unfavorable Food Stores With Incident HF, HFpEF, HFrEF, CHD, and Stroke

eTable 9. Association of High Frequencies to Unfavorable Food Stores With Incident HF, HFpEF, HFrEF, CHD, and Stroke

eFigure. Study Population Flow Diagram

This supplementary material has been provided by the authors to give readers additional information about their work.

© 2023 Zierath R et al. *JAMA Network Open.*

eMethods. Detailed Methods

Study Population

Second and third study visits for the JHS occurred between 2005 – 2008, and 2009 – 2013. The study was approved by the institutional review boards of Jackson State University, University of Mississippi Medical Center, and Tougaloo College. All participants provided written informed consent.

Frequency of Unhealthy Food Options

Geocoding analysis was performed at visit 1 where each participant was matched with the number of unfavorable food stores within a one-mile circular buffer of each participant's location (ArcVeiw 9.1, ESRI; Redlands, CA) as previously described in detail.[1-2]

Healthy Eating Index

At baseline, JHS participants completed a 158-item Food Frequency Questionnaire (FFQ) by trained interviewers.[3] The FFQ administered by JHS was adapted and validated from a 283-item FFQ created with data from two 24-hour dietary recalls collected from adults through the Mississippi Delta study[3-4] (r = 0.60 and 0.55 for men and women, respectively between the mean of repeated dietary recalls and the 158-item FFQ). The Healthy Eating Index (HEI) score was created based on data obtained from the FFQ. The score ranges from 0-100 and has 13 components divided into two categories: adequacy (Total Fruits, Whole Fruits, Total Vegetables, Greens and Beans, Whole Grains, Dairy, Total Protein Foods, Seafood and Plant Proteins, and Fatty Acids) where points are added for higher consumption; and moderation (Refined Grains, Sodium, Added Sugars, and Saturated Fats) where points are subtracted for higher consumption.[4]

Life's Simple 7 Diet Score

The Life's Simple 7 Diet Score was created based on how many of the following dietary criteria were met: 4.5 or more cups/day of fruits and vegetables; two or more 3.5 ounce servings/week of fish; 3 or more 1 ounce servings/day of whole grains; less than 1.5 grams/day of sodium; and less than 36 fluid ounces/week of sugar-sweetened beverages.[4]

Socioeconomic Status (SES) and Social Drivers of Health (SDOH)

The following income levels were assigned to each participant based on the US census poverty levels, taking into account both household income and family size: Poor, defined as having an income less than the poverty level; Lower-Middle, defined as having an income 1 to 1.5 times the poverty level; Upper-Middle, defined as having an income greater than 1.5 but less than 3.5 times the poverty level; and Affluent, defined as having an income equal to or greater than 3.5 times the poverty level.[5]

Perceived neighborhood violence was assessed with a 5-item questionnaire which assessed experiences with neighborhood fighting, sexual assault, and robbery. For each item, participants were asked to rate the frequency with which each experience occurred with a score of 1 (never) to 4 (often). Perceived neighborhood problems were assessed by having participants rate 1 (not really a problem) to 4 (very serious problem) for each item on a 6-item questionnaire assessing neighborhood noise, lack of park access, and litter. Neighborhood social cohesion was assessed with a 5-item questionnaire that had participants rate items from 1 (strongly disagree) to 4 (strongly agree) regarding trust in and willingness to help neighbors. Validated scales defined at the individual level for neighborhood problems, social cohesion and violence were created from a principal component analysis as previously described.[6-7]

Assessment of Pathway Biomarkers

B-type natriuretic peptide (BNP) concentration was measured by chemiluminescent immunoassay (ADVIA Centaur, Siemens). Renin levels were assessed by immunoradiometric assay. High-sensitivity C-reactive protein (hs-CRP) levels were measured by latex particle immunoturbidimetric assay (ITA, Roche Diagnostics). Leptin levels were analyzed by radioimmunoassay (LINCO Research, St. Charles, MI).[8]

Assessment of Clinical Outcomes

The criteria for definite or probable MI included ECG changes, chest pain symptoms, and cardiac enzyme levels. The criteria for fatal coronary disease events were comprised of combinations of the following: medical history or hospital information, chest pain symptoms, and underlying cause of death as listed on the death certificate.

© 2023 Zierath R et al. *JAMA Network Open.*

The criteria for cardiac procedure included medical record indications of angiography and any revascularization procedures.[9-10]

The minimum criteria for stroke included sudden or rapid onset of neurological symptoms lasting for greater than 24 hours or leading to death. Not included in stroke criteria were out-of-hospital stroke deaths not linked to hospitalization, hospitalized events with no medical chart available, any neurological symptoms that lasted less than 24 hours, or the lack of new neurological symptoms seen before or during hospitalization.[11]

Incident HF medical record abstraction and event adjudication was performed for hospitalization discharges with an International Classification of Disease (ICD) code 428 or an underlying cause of death I50, and included evaluation of either (A) radiographic findings consistent with HF or increased venous pressure or (B) autopsy finding of pulmonary edema/HF.[8] HF adjudication began on January 1, 2005.

© 2023 Zierath R et al. *JAMA Network Open.*

eTable 1. Baseline characteristics of the study population included and excluded from the study

	**Included in the study** *Mean (SD)*	**Excluded from the study** *Mean (SD)*	**P for difference**
	n=3024	N = 2282	
**Demographics**			
Age	54 (12)	56 (13)	<0.001
Sex (Male) *N (%)*	1037 (34)	902 (40)	<0.001
**Comorbidities**			
BMI	31.7 (7.1)	31.9 (7.4)	0.39
HTN *N (%)*	1585 (52)	1411 (62)	<0.001
Diabetes *N (%)*	623 (21)	619 (28)	<0.001
CKD *N (%)*	132 (4)	139 (6)	0.005
**Health Behaviors**			
Smoking Categorization *N (%)*			<0.001
Current smoker	328 (11)	365 (16)	
Quit < 12 months ago	37 (1)	27 (1)	
Quit > 12 months ago / Never smoked	2618 (88)	1843 (82)	
Physical Activity *N (%)*			<0.001
Poor	1378 (46)	1237 (54)	
Intermediate	1016 (34)	657 (29)	
Ideal	630 (21)	383 (17)	
**Diet and Stress**			
Perceived Stress	5.2 (4.3)	5.1 (4.4)	0.50
Life's Simple 7 Diet Score *N (%)*			0.70
Poor	1878 (68)	1373 (68)	
Intermediate	848 (31)	626 (32)	
Ideal	38 (1)	34 (2)	
Healthy Eating Index score	47.3 (10.5)	45.5 (10.1)	0.05
**Socioeconomic Status and Social Drivers of Health**			
Neighborhood % below poverty limit	0.23 (0.13)	0.25 (0.12)	<0.001
Income Categorization *N (%)*			<0.001
Poor	331 (13)	371 (20)	
Lower-Middle	579 (22)	520 (28)	
Upper-Middle	808 (31)	519 (28)	
Affluent	896 (34)	462 (25)	
Education Categorization *N (%)*			<0.001
< High school	383 (13)	590 (26)	
High school grad/GED	569 (19)	496 (22)	
Vocational school, trade school, college	2070 (68)	1178 (52)	
Lifetime Discrimination [0-9]	3.00 [1.00, 5.00]	3.00 [1.00, 4.00]	<0.001
Neighborhood problems	1.56 [1.37, 1.71]	1.58 [1.43, 1.72]	<0.001
Neighborhood violence	3.03 [2.93, 3.12]	3.01 [2.93, 3.09]	<0.001
Neighborhood social cohesion	1.26 [1.15, 1.32]	1.28 [1.18, 1.34]	<0.001
**Event Rate [95% CI per 100 PY]**			
Incident HF	2.9 [2.5 – 3.3]	4.3 [3.7 – 5.0]	

© 2023 Zierath R et al. *JAMA Network Open.*

Incident HFpEF	1.3 [1.1 – 1.6]	2.1 [1.7 – 2.6]	
Incident HFrEF	1.3 [1.0 – 1.6]	1.7 [1.4 – 2.2]	
Incident CHD	0.3 [0.3 – 0.4]	0.5 [0.4 – 0.6]	
Incident Stroke	0.3 [0.2 – 0.3]	0.4 [0.3 – 0.5]	

© 2023 Zierath R et al. *JAMA Network Open.*

eTable 2: Association of Economic Food Insecurity with Incident HF, HFpEF, HFrEF, CHD, and Stroke

Variable	N	Events (%)	Event rate (95% CI per 100 PY)	Unadjusted		Adjusted for demographics		Adjusted for demographics, comorbidities and SES	
				HR (95% CI)	*P* value	HR (95% CI)	*P* value	HR (95% CI)	*P* value
Incident HF									
Non-Food Insecure	2394	147 (7)	2.7 (2.3 – 3.2)	1.28 (0.93 – 1.78)	0.14	1.65 (1.15 – 2.36)	0.006	1.31 (0.88 – 1.97)	0.19
Food Insecure	630	48 (8)	3.6 (2.7 – 4.7)						
Incident HFpEF									
Non-Food Insecure	2394	70 (3)	1.3 (1.0 – 1.6)	1.01 (0.60 – 1.70)	0.96	1.26 (0.71– 2.24)	0.42	0.81 (0.42 – 1.53)	0.51
Food Insecure	630	18 (3)	1.3 (0.8 – 2.1)						
Incident HFrEF									
Non-Food Insecure	2394	63 (3)	1.2 (0.9 – 1.5)	1.54 (0.97 – 2.45)	0.07	2.01 (1.20 – 3.37)	0.008	2.07 (1.16 – 3.70)	0.014
Food Insecure	630	25 (4)	1.9 (1.3 – 2.7)						
Incident CHD									
Non-Food Insecure	2394	92 (4)	0.3 (0.3 – 0.4)	1.28 (0.85 – 1.93)	0.23	1.89 (1.20 – 3.00)	0.006	1.76 (1.06 – 2.91)	0.028
Food Insecure	630	31 (5)	0.4 (0.3 – 0.6)						
Incident Stroke									
Non-Food Insecure	2394	91 (4)	0.3 (0.2 – 0.4)	0.58 (0.31 – 1.06)	0.08	0.80 (0.41 – 1.56)	0.52	0.79 (0.38 – 1.64)	0.52
Food Insecure	630	13 (2)	0.2 (0.1 – 0.3)						

Demographics include age and sex; Co-morbidities and SES variables include hypertension, diabetes, Body Mass Index (BMI), estimated Glomerular Filtration Rate eGFR, income level, and educational attainment; HF - Heart Failure, HFpEF - Heart Failure with preserved Ejection Fraction, HFrEF - Heart Failure with reduced Ejection Fraction, CHD - Incident Coronary Heart Disease

© 2023 Zierath R et al. *JAMA Network Open.*

eTable 3: Association of Economic Food Insecurity with Incident HF, HFpEF, HFrEF, and CHD adjusted for diet quality and stress

Variable	N	Events (%)	Event rate (95% CI per 100 PY)	Adjusted for diet HEI score		Adjusted for diet LS7 score		Adjusted for stress		Adjusted for both (HEI + stress)	
				HR (95% CI)	*P* value	HR (95% CI)	*P* value	HR (95% CI)	*P* value	HR (95% CI)	*P* value
Incident HF											
Non-Food Insecure	2394	147 (7)	2.7 (2.3 – 3.2)	1.43 (0.94 – 2.18)	0.09	1.42 (0.93 – 2.17)	0.10	1.34 (0.89 – 2.02)	0.16	1.49 (0.97 – 2.28)	0.07
Food Insecure	630	48 (8)	3.6 (2.7 – 4.7)								
Incident HFpEF											
Non-Food Insecure	2394	70 (3)	1.3 (1.0 – 1.6)	0.86 (0.44 – 1.70)	0.67	0.85 (0.43 – 1.69)	0.65	0.80 (0.42 – 1.52)	0.49	0.88 (0.44 – 1.76)	0.71
Food Insecure	630	18 (3)	1.3 (0.8 – 2.1)								
Incident HFrEF											
Non-Food Insecure	2394	63 (3)	1.2 (0.9 – 1.5)	2.20 (1.21 – 3.99)	0.009	2.19 (1.21 – 3.98)	0.010	2.26 (1.24 – 4.12)	0.008	2.40 (1.30 – 4.43)	0.005
Food Insecure	630	25 (4)	1.9 (1.3 – 2.7)								
Incident CHD											
Non-Food Insecure	2394	92 (4)	0.3 (0.3 – 0.4)	1.77 (1.04 – 3.03)	0.036	1.76 (1.03 – 3.00)	0.04	2.00 (1.19 – 3.37)	0.009	2.04 (1.17 – 3.56)	0.012
Food Insecure	630	31 (5)	0.4 (0.3 – 0.6)								

Demographics include age and sex; Co-morbidities and SES variables include hypertension, diabetes, Body Mass Index (BMI), estimated Glomerular Filtration Rate eGFR, income level, and educational attainment; HF - Heart Failure, HFpEF - Heart Failure with preserved Ejection Fraction, HFrEF - Heart Failure with reduced Ejection Fraction, CHD - Incident Coronary Heart Disease

© 2023 Zierath R et al. *JAMA Network Open.*

eTable 4: Association of Economic Food Insecurity with Incident HF, HFpEF, HFrEF, CHD, and Stroke, Taking into Account the Competing Risk of Death

Variable	Unadjusted		Adjusted for demographics		Adjusted for demographics, comorbidities and SES	
	HR (95% CI)	*P* value	HR (95% CI)	*P* value	HR (95% CI)	*P* value
**Incident HF**						
Non-Food Insecure / Food Insecure	1.32 (0.96 – 1.82)	0.09	1.68 (1.19 – 2.36)	0.003	1.37 (0.92 – 2.02)	0.12
**Incident HFpEF**						
Non-Food Insecure / Food Insecure	1.03 (0.62 – 1.72)	0.90	1.27 (0.72– 2.22)	0.41	0.82 (0.43 – 1.58)	0.55
**Incident HFrEF**						
Non-Food Insecure / Food Insecure	1.59 (1.00 – 2.51)	0.05	2.03 (1.23 – 3.35)	0.006	2.16 (1.24 – 3.75)	0.006
**Incident CHD**						
Non-Food Insecure / Food Insecure	1.28 (0.85 – 1.93)	0.23	1.82 (1.16 – 2.86)	0.009	1.70 (1.04 – 2.77)	0.034
**Incident Stroke**						
Non-Food Insecure / Food Insecure	0.58 (0.32 – 1.06)	0.08	0.74 (0.38 – 1.43)	0.37	0.73 (0.36 – 1.47)	0.38

Demographics include age and sex; Co-morbidities and SES variables include hypertension, diabetes, Body Mass Index (BMI), estimated Glomerular Filtration Rate eGFR, income level, and educational attainment; HF - Heart Failure, HFpEF - Heart Failure with preserved Ejection Fraction, HFrEF - Heart Failure with reduced Ejection Fraction, CHD - Incident Coronary Heart Disease

© 2023 Zierath R et al. *JAMA Network Open.*

eTable 5: Association of Economic Food Insecurity with Incident HF, HFpEF, HFrEF, CHD, and Stroke censoring interim MI events

Variable	N	Events (%)	Event rate (95% CI per 100 PY)	Unadjusted		Adjusted for demographics		Adjusted for demographics, comorbidities and SES	
				HR (95% CI)	*P* value	HR (95% CI)	*P* value	HR (95% CI)	*P* value
Incident HF									
Non-Food Insecure	2386	145 (6)	2.7 (2.3 – 3.2)	1.29 (0.93 – 1.79)	0.12	1.65 (1.15 – 2.36)	0.006	1.31 (0.87 – 1.96)	0.20
Food Insecure	629	48 (8)	3.6 (2.7 – 4.7)						
Incident HFpEF									
Non-Food Insecure	2386	69 (3)	1.3 (1.0 – 1.6)	1.02 (0.61 – 1.72)	0.93	1.27 (0.72 – 2.25)	0.41	0.80 (0.42 – 1.53)	0.51
Food Insecure	629	18 (3)	1.3 (0.8 – 2.1)						
Incident HFrEF									
Non-Food Insecure	2386	62 (3)	1.2 (0.9 – 1.5)	1.55 (0.98 – 2.48)	0.06	1.99 (1.19 – 3.34)	0.009	2.04 (1.14 – 3.66)	0.02
Food Insecure	629	25 (4)	1.9 (1.3 – 2.7)						
Incident CHD									
Non-Food Insecure	2386	84 (4)	0.3 (0.2 – 0.3)	1.36 (0.90 – 2.06)	0.15	2.07 (1.30 – 3.31)	0.002	1.88 (1.13 – 3.13)9	0.02
Food Insecure	629	30 (5)	0.4 (0.3 – 0.5)						
Incident Stroke									
Non-Food Insecure	2386	89 (4)	0.3 (0.2 – 0.4)	0.59 (0.32 – 1.08)	0.09	0.80 (0.41 – 1.57)	0.52	0.79 (0.38 – 1.64)	0.52

© 2023 Zierath R et al. *JAMA Network Open.*

Food Insecure	629	13 (2)	0.2 (0.1 – 0.3)						

Demographics include age and sex; Co-morbidities and SES variables include hypertension, diabetes, Body Mass Index (BMI), estimated Glomerular Filtration Rate eGFR, income level, and educational attainment; HF - Heart Failure, HFpEF - Heart Failure with preserved Ejection Fraction, HFrEF - Heart Failure with reduced Ejection Fraction, CHD - Incident Coronary Heart Disease

© 2023 Zierath R et al. *JAMA Network Open.*

eTable 6: Association of Economic Food Insecurity with Incident HFpEF and HFrEF, with unknown EF assigned as HFpEF or HFrEF

Variable	N	Events (%)	Event rate (95% CI per 100 PY)	Unadjusted		Adjusted for demographics		Adjusted for demographics, comorbidities and SES	
				HR (95% CI)	*P* value	HR (95% CI)	*P* value	HR (95% CI)	*P* value
Incident HFpEF with unknown EF									
Non-Food Insecure	2394	84 (4)	1.6 (1.3 – 1.9)	1.09 (0.68 – 1.72)	0.73	1.38 (0.84– 2.28)	0.21	0.92 (0.52 – 1.61)	0.77
Food Insecure	630	23 (4)	1.7 (1.1– 2.6)						
Incident HFrEF with unknown EF									
Non-Food Insecure	2394	77 (3)	1.4 (1.1 – 1.8)	1.53 (1.00 – 2.33)	0.05	1.98 (1.24 – 3.15)	0.004	1.94 (1.15 – 3.29)	0.014
Food Insecure	630	30 (5)	2.2 (1.6 – 3.2)						

Demographics include age and sex; Co-morbidities and SES variables include hypertension, diabetes, Body Mass Index (BMI), estimated Glomerular Filtration Rate eGFR, income level, and educational attainment; HF - Heart Failure, HFpEF - Heart Failure with preserved Ejection Fraction, HFrEF - Heart Failure with reduced Ejection Fraction, CHD - Incident Coronary Heart Disease

© 2023 Zierath R et al. *JAMA Network Open.*

eTable 7: Baseline characteristics of study population overall and stratified by unfavorable food stores (greater or less than 2.5 food stores within 1 mile)

	**Overall** *Mean (SD)*	**Less than 2.5 unfavorable food stores within 1 mile** *Mean (SD)*	**More than 2.5 unfavorable food stores within 1 mile** *Mean (SD)*	**P value**
	n=3024	n=1506	n=1512	
**Demographics**				
Age	54 (12)	52 (12)	56 (13)	<0.001
Sex (Male) *N (%)*	1037 (34)	545 (36)	490 (32)	0.029
**Comorbidities**				
BMI	31.7 (7.1)	31.4 (6.9)	31.9 (7.3)	0.07
HTN *N (%)*	1585 (52)	709 (47)	874 (58)	<0.001
Diabetes *N (%)*	623 (21)	264 (18)	359 (24)	<0.001
CKD *N (%)*	132 (4)	62 (4)	70 (5)	0.49
**Health Behaviors**				
Smoking Categorization *N (%)*				0.014
Current smoker	328 (11)	141 (9)	187 (13)	
Quit < 12 months ago	37 (1)	16 (1)	21 (1)	
Quit > 12 months ago / Never smoked	2618 (88)	1337 (89)	1275 (86)	
Physical Activity *N (%)*				<0.001
Poor	1378 (46)	612 (41)	763 (50)	
Intermediate	1016 (34)	532 (35)	483 (32)	
Ideal	630 (21)	362 (24)	266 (18)	
**Diet and Stress**				
Perceived Stress	5.2 (4.3)	5.0 (4.0)	5.4 (4.6)	0.035
Life's Simple 7 Diet Score *N (%)*				0.68
Poor	1878 (68)	936 (68)	940 (68)	
Intermediate	848 (31)	433 (31)	415 (30)	
Ideal	38 (1)	17 (1)	21 (2)	
Healthy Eating Index score	47.3 (10.5)	47.1 (10.3)	47.4 (10.7)	0.56
**Socioeconomic Status and Social Drivers of Health**				
Neighborhood % below poverty limit	0.23 (0.13)	0.16 (0.10)	0.30 (0.11)	<0.001
Income Categorization *N (%)*				<0.001
Poor	331 (13)	125 (10)	205 (15)	
Lower-Middle	579 (22)	189 (15)	388 (29)	
Upper-Middle	808 (31)	378 (30)	428 (32)	
Affluent	896 (34)	561 (45)	334 (25)	
Education Categorization *N (%)*				<0.001
< High school	383 (13)	120 (8)	263 (17)	
High school grad/GED	569 (19)	230 (15)	337 (22)	
Vocational school, trade school, college	2070 (68)	1155 (77)	911 (60)	
Lifetime Discrimination [0-9]	3 [1, 5]	3 [2, 5]	3 [1, 4]	0.001
Neighborhood problems	1.56 [1.37, 1.71]	1.43 [1.37, 1.50]	1.66 [1.56, 1.79]	<0.001
Neighborhood violence	3.03 [2.93, 3.12]	3.07 [3.01, 3.18]	2.95 [2.90, 3.03]	<0.001

© 2023 Zierath R et al. *JAMA Network Open.*

Neighborhood social cohesion	1.26 [1.15, 1.32]	1.15 [1.14, 1.26]	1.29 [1.24, 1.34]	<0.001

© 2023 Zierath R et al. *JAMA Network Open.*

eTable 8: Association of Proximity to Unfavorable Food Stores with Incident HF, HFpEF, HFrEF, CHD, and Stroke

Variable	Events (%)	Unadjusted		Adjusted for demographics		Adjusted for demographics, comorbidities and SES	
		HR (95% CI)	*P* value	HR (95% CI)	*P* value	HR (95% CI)	*P* value
Incident HF							
Number of unhealthy food stores within one mile	195 (7)	1.06 (1.01 – 1.11)	0.028	1.02 (0.96 – 1.08)	0.51	1.02 (0.96 – 1.09)	0.47
Incident HFpEF							
Number of unhealthy food stores within one mile	88 (3)	1.07 (1.00 – 1.15)	0.05	1.00 (0.92 – 1.09)	1.00	1.00 (0.91 – 1.09)	0.94
Incident HFrEF							
Number of unhealthy food stores within one mile	88 (3)	1.04 (0.97 – 1.12)	0.31	1.03 (0.95 – 1.12)	0.41	1.05 (0.96 – 1.15)	0.27
Incident CHD							
Number of unhealthy food stores within one mile	123 (4)	1.05 (0.99 – 1.12)	0.08	1.02 (0.95 – 1.09)	0.61	1.00 (0.92 – 1.09)	0.96
Incident Stroke							
Number of unhealthy food stores within one mile	104 (4)	1.10 (1.03 – 1.17)	0.005	1.07 (0.99 – 1.15)	0.07	1.07 (0.99 – 1.16)	0.09

Demographics include age and sex; Co-morbidities and SES variables include hypertension, diabetes, Body Mass Index (BMI), estimated Glomerular Filtration Rate eGFR, income level, and educational attainment; HF - Heart Failure, HFpEF - Heart Failure with preserved Ejection Fraction, HFrEF - Heart Failure with reduced Ejection Fraction, CHD - Incident Coronary Heart Disease

© 2023 Zierath R et al. *JAMA Network Open.*

eTable 9: Association of High Frequencies of Unfavorable Food Stores with Incident HF, HFpEF, HFrEF, CHD, and Stroke

Variable	N	Events (%)	Event rate (95% CI per 100 PY)	Unadjusted		Adjusted for demographics		Adjusted for demographics, comorbidities and SES	
				HR (95% CI)	*P* value	HR (95% CI)	*P* value	HR (95% CI)	*P* value
Incident HF									
< 7.1 Food Stores	2743	172 (7)	2.8 (2.4 – 3.3)	1.31 (0.85 – 2.03)	0.22	1.24 (0.77 – 1.99)	0.39	1.15 (0.69– 1.91)	0.60
> 7.1 Food Stores	275	23 (9)	3.7 (2.4 – 5.5)						
Incident HFpEF									
< 7.1 Food Stores	2743	79 (3)	1.3 (1.0 – 1.6)	1.12 (0.56 – 2.23)	0.75	0.99 (0.46 – 2.17)	0.99	0.85 (0.37 – 1.98)	0.71
> 7.1 Food Stores	275	9 (3)	1.4 (0.7 – 2.8)						
Incident HFrEF									
< 7.1 Food Stores	2743	75 (3)	1.2 (1.0 – 1.5)	1.69 (0.94 – 3.06)	0.08	1.68 (0.88 – 3.19)	0.11	1.70 (0.85 – 3.37)	0.13
> 7.1 Food Stores	275	13 (5)	2.1 (1.2 – 3.6)						
Incident CHD									
< 7.1 Food Stores	2743	112 (4)	0.3 (0.3 – 0.4)	1.01 (0.55 – 1.89)	0.96	0.86 (0.42 – 1.78)	0.69	0.80 (0.37 – 1.75)	0.58
> 7.1 Food Stores	275	11 (4)	0.3 (0.2 – 0.6)						
Incident Stroke									
< 7.1 Food Stores	2743	92 (4)	0.2 (0.1 – 0.3)	1.37 (0.73 – 2.57)	0.33	1.58 (0.84 – 3.00)	0.16	1.63 (0.85 – 3.13)	0.14

© 2023 Zierath R et al. *JAMA Network Open.*

> 7.1 Food Stores	275	12 (5)	0.4 (0.2 – 0.6)						

Demographics include age and sex; Co-morbidities and SES variables include hypertension, diabetes, Body Mass Index (BMI), estimated Glomerular Filtration Rate eGFR, income level, and educational attainment; HF - Heart Failure, HFpEF - Heart Failure with preserved Ejection Fraction, HFrEF - Heart Failure with reduced Ejection Fraction, CHD - Incident Coronary Heart Disease

© 2023 Zierath R et al. *JAMA Network Open.*

eFigure: Study population flow diagram

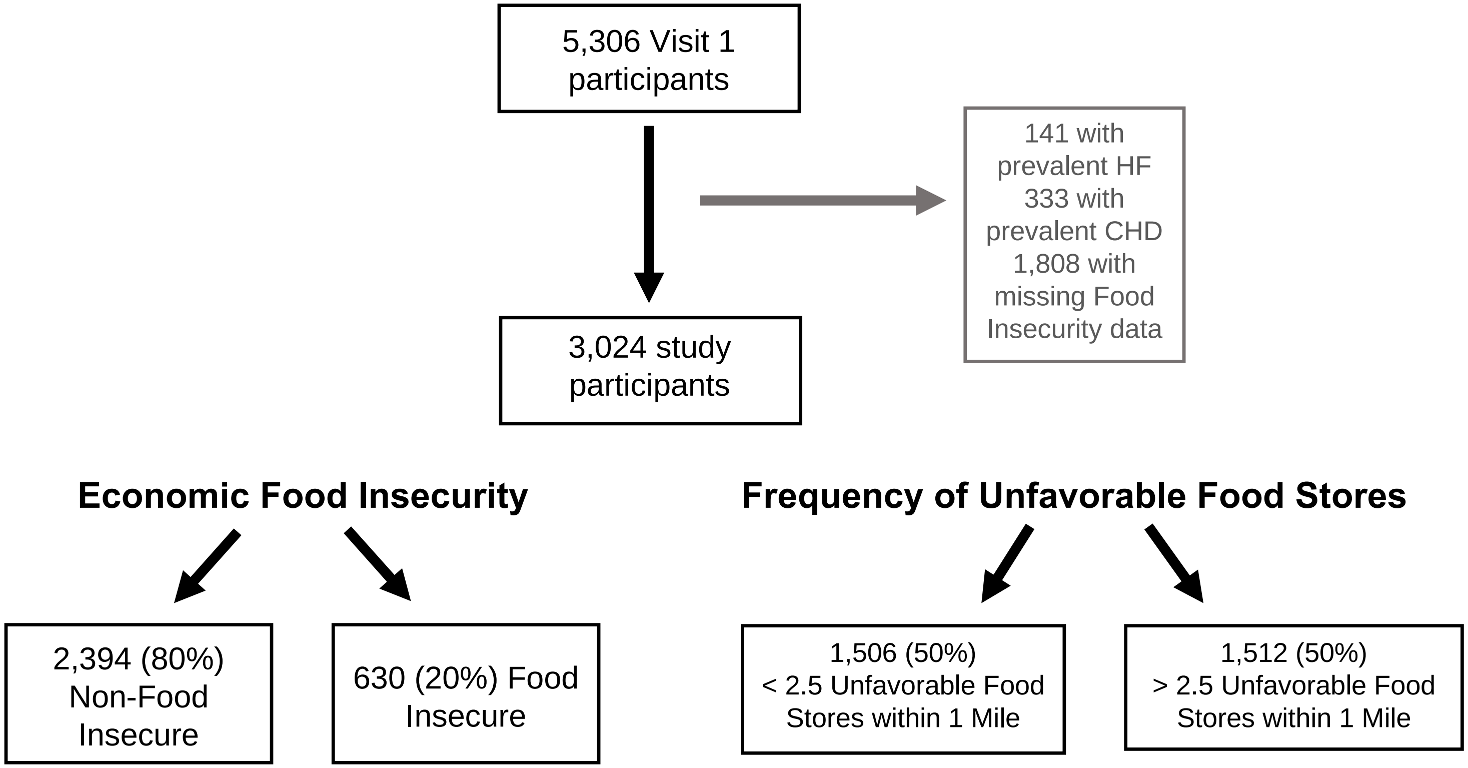

© 2023 Zierath R et al. *JAMA Network Open.*

eReferences

1. Robinson JC, Wyatt SB, Hickson D et al. Methods for retrospective geocoding in population studies: the Jackson Heart Study. J Urban Health 2010;87:136-50.

2. Hickson DA, Diez Roux AV, Smith AE et al. Associations of fast food restaurant availability with dietary intake and weight among African Americans in the Jackson Heart Study, 2000-2004. Am J Public Health 2011;101 Suppl 1:S301-9.

3. Carithers TC, Talegawkar SA, Rowser ML et al. Validity and calibration of food frequency questionnaires used with African-American adults in the Jackson Heart Study. J Am Diet Assoc 2009;109:1184-1193.

4. Carithers T, Dubbert PM, Crook E et al. Dietary assessment in African Americans: methods used in the Jackson Heart Study. Ethn Dis 2005;15:S6-49-55.

5. Sims M, Diez-Roux AV, Gebreab SY et al. Perceived discrimination is associated with health behaviours among African-Americans in the Jackson Heart Study. J Epidemiol Community Health 2016;70:187-94.

6. Gebreab SY, Hickson DA, Sims M et al. Neighborhood social and physical environments and type 2 diabetes mellitus in African Americans: The Jackson Heart Study. Health Place 2017;43:128-137.

7. Mujahid MS, Diez Roux AV, Morenoff JD, Raghunathan T. Assessing the measurement properties of neighborhood scales: from psychometrics to ecometrics. Am J Epidemiol 2007;165:858-67.

8. Fox ER, Samdarshi TE, Musani SK et al. Development and Validation of Risk Prediction Models for Cardiovascular Events in Black Adults: The Jackson Heart Study Cohort. JAMA Cardiol 2016;1:15-25.

9. Keku E, Rosamond W, Taylor HA, Jr. et al. Cardiovascular disease event classification in the Jackson Heart Study: methods and procedures. Ethn Dis 2005;15:S6-62-70.

10. White AD, Folsom AR, Chambless LE et al. Community surveillance of coronary heart disease in the Atherosclerosis Risk in Communities (ARIC) Study: methods and initial two years' experience. J Clin Epidemiol 1996;49:223-33.

11. Rosamond WD, Folsom AR, Chambless LE et al. Stroke incidence and survival among middle-aged adults: 9-year follow-up of the Atherosclerosis Risk in Communities (ARIC) cohort. Stroke 1999;30:736-43.

© 2023 Zierath R et al. *JAMA Network Open.*
