# Supplementary material for: Measures of Food Inadequacy and Cardiovascular Disease Risk in Black Individuals in the US From the Jackson Heart Study
Source: JAMA Netw Open. 2023 Jan 23;6(1):e2252055. doi: 10.1001/jamanetworkopen.2022.52055 (PMC9871801; doi:10.1001/jamanetworkopen.2022.52055)
Supplement: Supplement 2. — Data Sharing Statement [file jamanetwopen-e2252055-s002.pdf]

Data Sharing Statement

Zierath. Measures of Food Inadequacy and Cardiovascular Disease Risk in Black Individuals in the US From the Jackson Heart Study. *JAMA Netw Open*. Published January 23, 2023. doi:10.1001/jamanetworkopen.2022.52055

Data
Data available: No
